# Twenty-Five Years as a Hospital Secretary

**Published:** 1907-02-23

**Authors:** 


### TWENTY-FIVE YEARS AS A HOSPITAL SECRETARY.

### PRESENTATION TO MR. P. J. MICHELLI, C.M.G.

Mr. P. Michelli was the recipient of a handsome presentation at a complimentary dinner given on February 15 at the Trocadero Restaurant in commemoration of his having had conferred upon him the honour of C.M.G. and his having completed the twentieth year of his office as Secretary of the Seamen's Hospital Society. Mr. Perceval Nairne, the Chairman of the Society, occupied the chair, and amongst those present were Sir Henry Burdett, Sir William Treacher, Sir William Bennett, Sir Patrick and Lady Manson and Miss Manson, Sir John and Lady Craggs, the Consul-General for Austria-Hungary, Dr. Arthur Lathom, Dr. and Mrs. Guthrie Rankin, Messrs. Thomas Ryan (Secretary of St. Mary's Hospital), T. Hayes (clerk to St. Bartholomew's Hospital), and Clare Melhado (Secretary-Superintendent of the Middlesex Hospital).

The presentation took the form of an elegant silver gilt William IV. centrepiece dated 1836, weighing 95 oz., on the base of which was the inscription : "Presented to Pietro Michelli, Esqre., C.M.G., by his Colleagues and Friends. 15th February, 1907."

After the toast of "The King," the Chairman proposed the health of "Our Guest." The honour conferred by his Majesty upon Mr. Michelli had been a matter of the highest gratification not only to his family but to all who had had the opportunity of working with him in the various branches of his work for so many years. (Applause.) Brought up in the Cove of Cork, Mr. Michelli had a natural predilection for the sea and ships, and in his early days, serving in the office of his father, the Consul for Austria, at Cork, he had become more closely connected still with ships and seamen. Thirty years ago Mr. Michelli became the Steward of the Seamen's Hospital at Greenwich, a post which had led him, as it had led a good many of his successors, to a high position in the hospital world. Since that time, with the exception of the five years from 1882 to 1887, when he held the very responsible position of Secretary to St. Mary's Hospital, Mr. Michelli has been connected with the Seamen's Hospital. All those who had been engaged in the internal work of the hospital knew what devotion he had shown to its interests and to what a very great extent the hospital had been indebted to him. (Hear, hear.) But Mr. Michelli had in addition made himself a name and a position in the hospital world which was certainly a very high one. Years ago he served as the Honorary Secretary of Lord Sandhurst's Committee on the Metropolitan Hospitals, later on he was Hon. Secretary of the Hospitals Association, recently he had been, and the speaker believed was still, a member of the Special Committee appointed by the Council of King Edward's Hospital Fund to consider the simplification and assimilation of hospital accounts and statistics, and besides this he had been the President of the Hospital Officers' Association, and was at that moment the President of the Hospital Officers' Club. (Applause.) At the time when Mr. Michelli joined the staff of the hospital, the *Dreadnought* Hospital was the sole institution connected with the Society. Since that time the Society had spread and now included a branch hospital between the Victoria and Albert Docks, dispensaries at the East and West India Docks and at Gravesend, the London School of Tropical Medicine, and the London School of Clinical Medicine. When they considered what a great addition these developments had been to the secretarial duties, they would see that the services Mr. Michelli had rendered to the Seamen's Hospital and to the hospital world at large were of no mean character. (Applause.) During the whole of this time his relations with the staff of the hospital had been of the most cordial nature—the whole of the professional, as well as the civil, staff looked to Mr. Michelli to bring them through any difficulty, and he had done this with the most remarkable success. In the name of Mr. Michelli's friends and associates in hospital work, he had to ask his acceptance of an antique centrepiece, which they hoped might be a sort of memento for his family of the appreciation of the Seamen's Hospital Society and of those friends who had been associated with him in work which he had done so unfailingly and ungrudgingly in the cause of hospitals at large. (Applause.) As his friends knew that a gift to him personally would only be half a gift, he (the speaker) had to ask Mr. Michelli's acceptance, on behalf of Mrs. Michelli, of a diamond bracelet, which they felt no doubt Mrs. Michelli would look upon with some pride as a recognition of a very distinguished period in the professional and social life of her husband. (Applause.) They all wished Mr. and Mrs. Michelli long life and continued prosperity, and hoped that they might enjoy their own society and the distinctions which it had pleased his Majesty the King of Great Britain and Ireland, and his Majesty the King of Sweden, to confer upon him with those of all the other Sovereigns who might decorate Mr. Michelli in time to come. (Loud applause.)

Mr. Michelli (who was greeted with loud and continued applause) returned thanks. He said he thought that it must fall to the lot of but few secretaries to be honoured as he was honoured that evening. To much fewer must the privilege be given to be able to recognise the services they had received not only at the hands of the Board they served, but at the hands of such distinguished members of the medical profession as constituted the medical staff of the Seamen's Hospital Society. After referring to the assistance he had received from the chairmen he had worked under and the Board of Management under whom it was a privilege to serve, and to whose enterprise and public spirit was due the success of the hospital and the establishment of the two schools of medicine which had been referred to by the Chairman, Mr. Michelli said that another matter which had conduced to the success of the hospital was the absolute confidence which had existed between the Board of Management and the medical staff. Whilst he recognised his indebtedness to those he served under, he would like to be allowed to say how much he owed to those who served with and under him; and he was not unmindful of what he owed to his colleagues in the hospital world. Most gratifying, too, to him was it to see present that evening Sir Henry Burdett, a predecessor of his in the position he held, through whose courtesy he owed his first insight into hospital life, and who, in all the years which had followed, had shown him many and great kindnesses. After mentioning that there were no fewer than nine men acting as secretaries to hospitals at the present time who had served their apprenticeship at the *Dreadnought* Hospital, Mr. Michelli returned his most cordial thanks on behalf of himself and his wife for the honour which had been done him, and added that that evening had been made doubly dear to him by their associating Mrs. Michelli with himself on that occasion. (Applause.)

Sir Henry Burdett, in proposing the health of "The Society," referred in feeling terms to the Chairman, Mr. Nairne, with whom he had been associated in hospital affairs for thirty-four years. He thought that Mr. Nairne had done a work which ought to be definitely and publicly recognised, because it amounted to so much, to so many interests, and to so many people not only in the country but throughout the British Empire. (Applause.) Mr. Nairne had been the master mind which had directed most of the developments which had made for the success of the Society, and he hoped—because he believed that public recognition was the best aid to progress and the best encouragement to the best work—that before a very long time had elapsed there might be some public recognition of this quiet, modest, firm, great man, who, because of his retiring nature and because of his strength, had been far too little known and far too little recognised. (Applause.) Finally, he would like to see by the centenary of the Society in 1921 that they had raised the money to put up the finest modern hospital that, with all their experience and knowledge, they were capable of producing, and that most of those present that evening would have the privilege of taking part at the opening of this modern hospital devoted to the care and the comfort of the seamen of all nations. (Applause.)

Sir Patrick Manson responded on behalf of the London School of Tropical Medicine, and Sir William Bennett on behalf of the School of Clinical Medicine.

Sir William Treacher, in proposing the health of "The Chairman," endorsed what Sir Henry Burdett had said with reference to a public recognition of the great services rendered by Mr. Nairne to the Seamen's Hospital.

The Chairman having briefly returned thanks,

Sir Henry Burdett proposed "The Ladies" and the health of the Honorary Secretary to the dinner—Mr. Kenneth Steele—whose reply concluded the proceedings.

